# Gait Pattern and Motor Performance During Discrete Gait Perturbation in Children With Autism Spectrum Disorders

**DOI:** 10.3389/fpsyg.2018.02530

**Published:** 2018-12-11

**Authors:** Emilia Biffi, Cristina Costantini, Silvia Busti Ceccarelli, Ambra Cesareo, Gian Marco Marzocchi, Maria Nobile, Massimo Molteni, Alessandro Crippa

**Affiliations:** ^1^Scientific Institute, IRCCS E. Medea, Lecco, Italy; ^2^Department of Psychology, University of Milano-Bicocca, Milan, Italy; ^3^Department of Electronics, Information, and Bioengineering, Politecnico di Milano, Milan, Italy

**Keywords:** autism spectrum disorder, gait analysis, motor adaptation, dual-belt treadmill, virtual reality

## Abstract

Quantitative evaluation of gait has been considered a useful tool with which to identify subtle signs of motor system peculiarities in autism spectrum disorder (ASD). However, there is a paucity of studies reporting gait data in ASD as well as investigating learning processes of locomotor activity. Novel advanced technologies that couple treadmills with virtual reality environments and motion capture systems allows the evaluation of gait patterns on multiple steps and the effects of induced gait perturbations, as well as the ability to manipulate visual and proprioceptive feedbacks. This study aims at describing the gait pattern and motor performance during discrete gait perturbation of drug-naïve, school-aged children with ASD compared to typically developing (TD) peers matched by gender and age. Gait analysis was carried out in an immersive virtual environment using a 3-D motion analysis system with a dual-belt, instrumented treadmill. After 6 min of walking, 20 steps were recorded as baseline. Then, each participant was exposed to 20 trials with a discrete gait perturbation applying a split-belt acceleration to the dominant side at toe-off. Single steps around perturbations were inspected. Finally, 20 steps were recorded during a post-perturbation session. At baseline, children with ASD had reduced ankle flexion moment, greater hip flexion at the initial contact, and greater pelvic anteversion. After the discrete gait perturbation, variations of peak of knee extension significantly differed between groups and correlated with the severity of autistic core symptoms. Throughout perturbation trials, more than 60% of parameters showed reliable adaptation with a decay rate comparable between groups. Overall, these findings depicted gait peculiarities in children with ASD, including both kinetic and kinematic features; a motor adaptation comparable to their TD peers, even though with an atypical pattern; and a motor adaptation rate comparable to TD children but involving different aspects of locomotion. The platform showed its usability with children with ASD and its reliability in the definition of paradigms for the study of motor learning while doing complex tasks, such as gait. The additional possibility to accurately manipulate visual and proprioceptive feedback will allow researchers to systematically investigate motor system features in people with ASD.

## Introduction

Autism spectrum disorder (ASD) is a highly heterogeneous neurodevelopmental disorder characterized by persistent social impairment, communication abnormalities, and restricted and repetitive behaviors (American Psychiatric Association, 2013). Alongside socio-communicative symptoms, recognizing the motor difficulties frequently associated with ASD is of great importance, as they could impact the quality of life and are critical to prospective social development (Lai et al., 2014). Even in the earliest descriptions of the disorder (Kanner, 1943; Asperger, 1944), widespread abnormalities of movement have been described, including atypical postural control, gait, reach-to-grasp movements, and gross and fine motor control. Among these domains, the study of gait in people with ASD has drawn growing interest in the last decades. The acquisition and development of locomotor ability are crucial for children’s exploration of the surrounding space and for their ability to consent to a successful interaction with the environment. As a consequence, gait represents a key activity for the progressive development of the children’s communication.

Given these considerations, the systematic evaluation of locomotor activity, based on quantitative methods such as kinematic analysis, has been considered to be a useful tool with which to detect signs of motor system dysfunction in people with ASD (Nayate et al., 2005), and to explore possible neural correlates of motor anomalies. Moreover, retrospective analyses of home videos of infants and toddlers who were later diagnosed with ASD have suggested that abnormal gait patterns may precede social and communicative difficulties (Teitelbaum et al., 1998; Esposito and Venuti, 2008; Esposito et al., 2011). Last, a recent survey of clinicians who diagnose ASD showed that atypical gait is one of the behaviors recurrently associated with a “frank” manifestation of the condition (De Marchena and Miller, 2017).

Ever since the first quantitative study of gait in children with ASD (Vilensky et al., 1981), a number of empirical studies on gait activity have been conducted with mixed findings in the types of locomotor anomalies described in people with ASD (see Kindregan et al., 2015, for a recent review). The causes of these puzzling results might be various, as the studies differed in many important methodological aspects, such as the technologies used, sample sizes, and participants’ ages. At this stage of the scientific literature, there is a lack of studies reporting gait data in people with ASD and investigating the learning processes of locomotor activity.

Novel advanced technologies that combine treadmills with virtual reality (VR) environments and motion capture systems allow the evaluation of gait patterns on multiple steps and the effects of induced gait perturbations, as well as the manipulation of visual and proprioceptive feedback. This study aimed to describe the gait pattern of drug-naïve, school-aged children with ASD compared to their typically developing (TD) peers using the Gait Real-time Analysis Interactive Lab (GRAIL), a multi-sensor platform based on immersive VR, with an instrumented dual-belt treadmill that acquires and processes kinetic and kinematic data in real time. To the best of our knowledge, no studies have yet employed this innovative, integrated system to examine the gait activity in patients with neurodevelopmental disorders. Based on the findings of studies of overground gait (see, for example, Rinehart et al., 2006; Calhoun et al., 2011; Nobile et al., 2011; Pauk et al., 2017), we expected children with ASD to have gait peculiarities with respect to spatio-temporal, kinetic, and kinematic parameters, even though walking on a treadmill can slightly affect these gait parameters (Van der Krogt et al., 2014). Furthermore, we intended to explore the relationship between potential differences in gait parameters and core features of ASD. Last, a second main goal of this work was to examine the gait adaptation during multiple discrete perturbations in the same cohort of participants. The discrete perturbation was applied using the GRAIL split-belt treadmill, with the independent belt under the dominant leg suddenly accelerating at a single toe-off phase of 20 gait trials. It has already been shown that the adaptation to similar split-belt perturbations might rely on the cerebellum (Morton and Bastian, 2006). Decades of anatomical and imaging data (Bauman and Kemper, 1985; Courchesne et al., 1988; Becker and Stoodley, 2013) suggested that the cerebellum could be primarily implicated in ASD, with cognitive and behavioral outcomes beyond the manifestations of the motor domain. With particular reference to gait, evidence from previous studies on ASD are consistent with a cerebellar involvement (Vilensky et al., 1981; Hallett and Massaquoi, 1993; Ambrosini et al., 1998; Rinehart et al., 2006; Nobile et al., 2011). To date, no studies have investigated this type of adaptation to discrete gait perturbations in children with ASD. Based on the literature regarding upper limb motor learning and adaptation in people with ASD (Haswell et al., 2009; Izawa et al., 2012; Marko et al., 2015), we expected children with ASD to have a slower rate or an atypical pattern of adaptation.

## Materials and Methods

### Participants

Children in the ASD group were recruited at the Child Psychopathology Unit of Scientific Institute, IRCCS Eugenio Medea (Lecco, Italy). Inclusion criteria included having a clinical diagnosis of ASD, having a full-scale IQ (FSIQ) of 80 or above, and being aged between 7 and 12. Exclusion criteria included using any stimulant or non-stimulant medication that affects the central nervous system, having an identified genetic disorder, having vision or hearing problems, and suffering from chronic or acute medical illness.

All participants in the clinical group had been previously diagnosed according to the Diagnostic and Statistical Manual of Mental Disorders (4th ed., text rev.) (American Psychiatric Association, 2000) at admission by a medical doctor specialized in child neuropsychiatry with expertise in autism. The diagnoses were then confirmed using the Autism Diagnostic Observation Schedule (ADOS) (Lord et al., 2002). Children in the healthy control group (TD) were recruited by local pediatricians, were from kindergartens in the vicinity of our institute, and were gender- and age-matched to the clinical sample from the TD population. The TD children had no previous history of social/communicative disorders, developmental abnormalities, or medical disorders with central nervous system implications.

The study was explained to both the children and their parent(s) or caregiver(s), and all of the participants’ legal guardians signed the informed written consent before the children participated. The research received approval from the ethic committee of our institute “Comitato Etico IRCCS E. Medea – Sezione Scientifica Associazione La Nostra Famiglia” and was therefore performed in accordance with the ethical standards set forth in the 1964 Declaration of Helsinki and its later amendments.

### Experimental Protocol

Participants were evaluated with the following measures. At least five subtests of the WISC-IV [Vocabulary, Similarities, Block Design, and Matrix Reasoning, Picture Concepts; (Wechsler, 2012)] were administered to all children to determine the general IQ level (Crawford et al., 2010). All participants were required to have estimated FSIQ of 80 or above. Perceptual reasoning index was used as the measure of intellectual reasoning ability to match the two groups of participants. Being a measure of non-verbal abilities, the perceptual reasoning index may better estimate cognitive abilities in subject with ASD than the FSIQ (Mottron, 2004). Parents completed the Social Responsiveness Scales (SRS) (Constantino and Gruber, 2005) to measure general autistic symptoms/traits and behavior difficulties across participants, and the Social Communication Questionnaire–Lifetime (Rutter et al., 2003) to check for ASD symptoms in the TD children. Last, data on parental employment were used as a measure of socioeconomic status and coded according to the Hollingshead 9-point scale for parental occupation (Hollingshead, 1975).

The motor skills of the participants were assessed using the Movement Assessment Battery for Children 2 (MABC2) (Henderson et al., 2007). The MABC2 consists of eight subtests that evaluate three components of motor proficiency: manual dexterity, ball skills, and static and dynamic balance. For the MABC2, higher scores are indicative of better motor performance. In addition, parents completed the Developmental Coordination Disorder Questionnaire (DCDQ) (Wilson et al., 2007). The DCDQ is a 15-item questionnaire that investigates gross and fine motor skill impairments. The DCDQ yields a raw total score (score range 15–75); higher scores indicate better motor functioning as rated by the parents.

**FIGURE 1:**
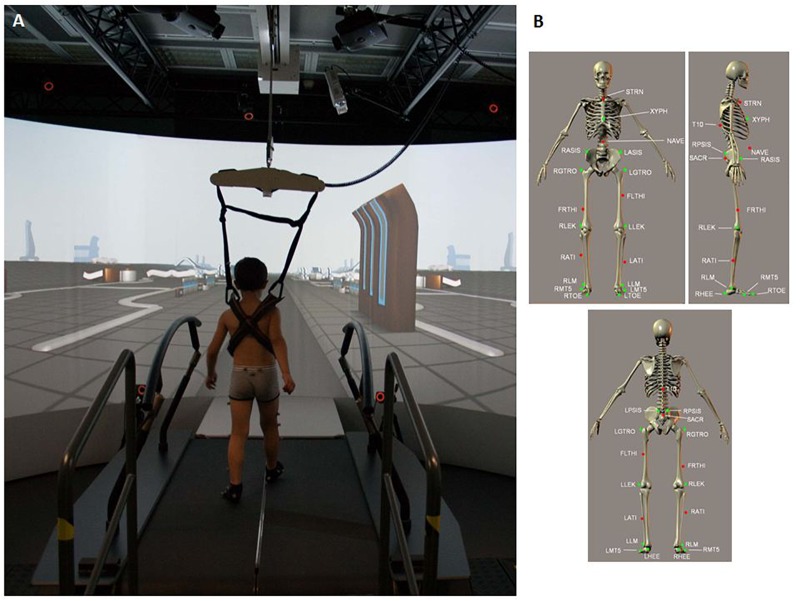
**(A)** Child walking on the GRAIL system during the session. **(B)** 25 marker set. T10, On the 10th thoracic vertebrae. SACR, On the sacral bone; NAVE, On the navel; XYPH, Xiphoid process of the sternum; STRN, On the jugular notch of the sternum; LASIS, Left anterior superior iliac spine; RASIS, Right anterior superior iliac spine; LPSIS, Left posterior superior iliac spine; RPSIS, Right posterior superior iliac spine; LGTRO, On the center of the left greater trochanter; FLTHI, On 1/3 on the line between the LGTRO and LLEK; LLEK, On the lateral side of the joint axis; LATI, On 2/3 on the line between the LLEK and LLM; LLM, The center of left lateral malleolus.

The gait features of the participants were evaluated in terms of kinematics and kinetics using the GRAIL (Motekforce Link; Figure 1A). The GRAIL is equipped with an instrumented dual-belt treadmill that integrates 16-channel force plates (sample frequency of 1000 Hz) and is placed over a two-degrees-of-freedom motion frame. The motion frame can translate in the longitudinal and lateral direction, and each belt of the treadmill can be independently accelerated or decelerated to assess compensatory strategies and to investigate dynamic stability. Around the system is motion-capture equipment (Vicon system) with 10 optoelectronic cameras (sample frequency of 100 Hz) to acquire kinematic data and 3 video cameras. The system is surrounded by a 180° cylindrical projection screen where VR environments are projected with an optic flow that is synchronized to the speed of the treadmill. Kinematic and kinetic data of multiple steps are processed in real time using the 25-marker Human Body Model (Van den Bogert et al., 2013) (Figure 1B) and saved in a.mox file.

The participants were instructed to walk at their own natural speed for 6 min in the mediolateral middle of the treadmill, placing one foot on each separate belt. During this habituation period, the speed of the belts was real-time adjusted to meet each child’s time-varying walking pace. After this 6-min period of adaptation, a 20-step trial was recorded as baseline (T0). Subsequently, each participant was exposed to 20 trials with a discrete gait perturbation: after a random number of steps, a single perturbation was applied to the dominant side at toe-off, using a split-belt acceleration. Single steps around perturbations (before, during, and after) were recorded. Finally, at the end of the perturbed trials, we recorded 20 steps as a post-perturbation trial (T1).

### Data Analysis and Statistics

Between-group differences on the demographic variables, questionnaires, and cognitive and motor measures were analyzed using an independent-samples *t*-test, according to the distributional nature of the data.

Concerning gait data, the Gait Offline Analysis Tool was used to load the recorded.mox file, to perform a filtering with a 2nd-order Butterworth filter (cut-off frequency equal to 6 Hz), to automatically recognize and exclude strides with foot placement on both belts, to align gait traces at the initial contact (IC), and to save them in a.csv file.

Then, for each subject, a custom software developed in MATLAB (The MathWorks^®^) was run to perform the following actions:

(a) Step by step extraction of gait features from the ankle, knee, hip and pelvic waveforms. The gait parameters, computed for the left and the right side, were: the stance period, computed as a percentage of the gait cycle (it begins with initial contact of foot and ends at toe off of the same limb), the step length, and the walking speed as spatio-temporal parameters; the peak flexion moments for the ankle, knee, and hip joints for kinetic evaluations; the flexion at the IC, the range of motion (ROM) in flexion in the sagittal plane, and the peak of extension for the three joints; and the pelvic tilt at the IC and its mean value during the gait cycle.

(b) Check for normality of each gait feature by means of the Shapiro–Wilk test.

(c) If normal, calculation of the mean value of each gait parameter over *N* steps recorded. In contrast, median values are computed.

**FIGURE 2:**
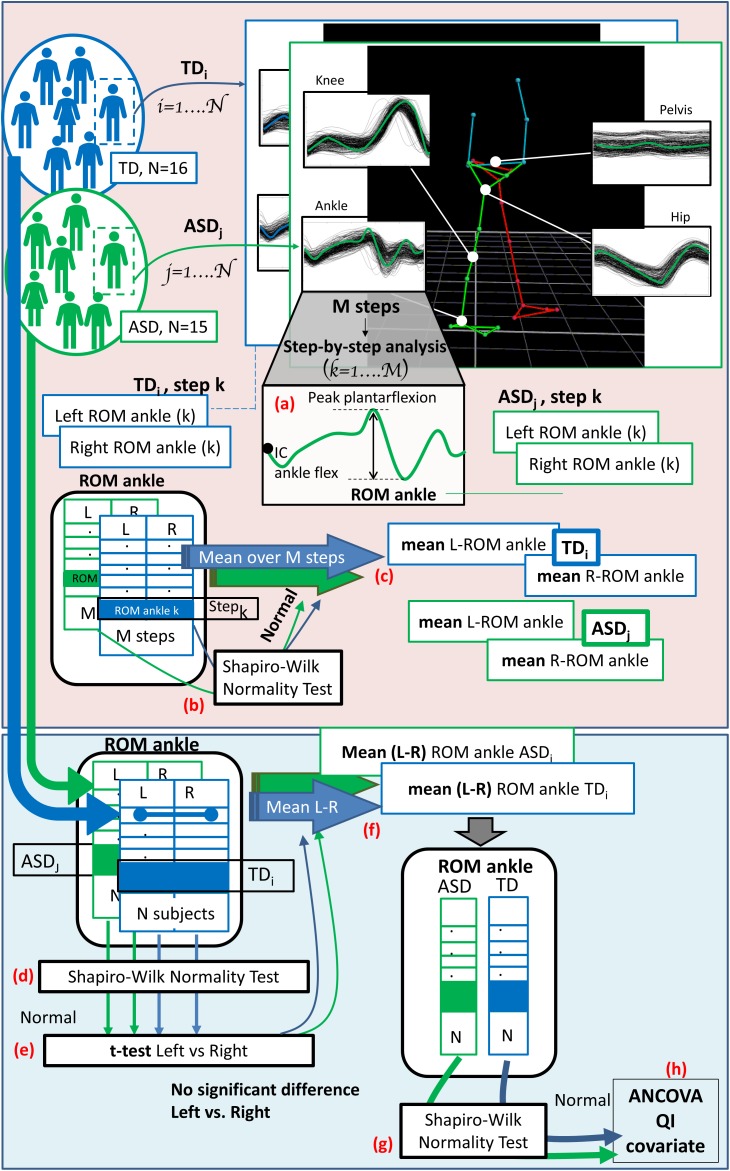
The diagram shows the entire data analysis process from the step-by-step gait features extraction to the ANCOVA analysis, considering a single exemplifying gait feature (the range of motion of the ankle: “ROM ankle”). Light pink panel shows the single subject analysis (run subject per subject). **(a)** At first the gait features are extracted for each step, both for the right and left side. Then, **(b)** normality is checked considering the values of all the M steps and, if verified, **(c)** the mean value over all the steps is computed, for both the right and left side. Light blue panel depicts group analysis (run across all subject of TD and ASD group). Left and right (mean) values for each subject are collected and **(d)** normality is tested; **(e)** *t*-test or Wilcoxon test, depending on the data distribution, is performed to check for differences between left and right side. **(f)** When difference are not significant the mean value between left and right sides is computed for each subject. **(g)** Shapiro–Wilk test is run to check for normality within groups and, since verified, **(h)** ANCOVA analysis with QI as covariate is performed.

Then, left and right (mean) values of each gait feature, related to each subject were organized in tables (one for the ASD and one for the TD group) and the following operations were run:

(d) Check for normality of each gait feature within each group for the left and the right side by means of the Shapiro–Wilk test;

(e) *T*-test or Wilcoxon test, depending on the data distribution, to check for differences between left and right side.

(f) When no or few differences between sides were detected, the right and the left limb were pooled and the mean value of each parameter for each patient was computed.

(g) Shapiro–Wilk test to check for normality of each gait feature (left and right mean values) in the two groups. This procedure was performed for the T0 and T1 trials. A single-step analysis of the cycles before, during, and after split-belt acceleration was performed for the 20 perturbed trials.

(h) The final step was, once verified the normality of the data distribution, to run an analysis of covariance (ANCOVA), with the IQ as covariate, to compare the two groups of children across all experimental gait measures at T0. Figure 2 shows the data analysis flow, from the step-by-step gait features extraction to the ANCOVA analysis.

The same analysis was also run on the normalized variation between T0 and T1, that was computed as follows (equation 1):

(1) $$\begin{array}{lll} {\mathrm{Delta}}_{\text{i}-\text{parameter}} & = & \left(T1_{\text{i}-\text{parameter}}-T0_{\text{i}-\text{parameter}}\right)\\ & & /\;T0_{\text{i}-\text{parameter}} \end{array}$$

For gait parameters that showed significant differences between groups, a regression analysis between gait data and ASD symptom measures (ADOS and SRS) was performed, and residuals were compared with bivariate Pearson correlations to identify associations.

To compare the adaptation rate across groups when the participants were exposed to a perturbation, we examined whether a linear function (equation 2) fit the data derived for each variable on all the steps before, during, and after the perturbation (Rand et al., 2004).

(2) y=b1+b2*x

where *b*1 is the slope intercept; *b*2 is the angular coefficient of the slope, thus representing the adaptation rate; and x is the trial number.

Furthermore, R^2^ and b2 values that fit significantly were compared between groups with the independent-samples *t*-test to investigate possible differences in learning. Moreover, considering only the variables that showed a significant fitting in at least in one group, T0 and T1 data were compared within the group with a paired sample *t*-test. The significance level was two tailed, *p* < 0.05, for all the analyses. Because this study was exploratory, no correction was applied for the family-wise error rate.

## Results

Fifteen children with ASD (mean age: 9.8 ± 1.6 years; 14 males, 1 female) and sixteen TD children (mean age: 10.0 ± 1.3 years; 15 males, 1 female), matched by gender and age, were enrolled. All participants were Caucasian, had normal or corrected-to-normal vision, and were not taking any medication. One children with ASD with an FSIQ of 76 was included on the basis of discrepant subscores and having a Perceptual Reasoning Index of 80.

As shown in Table 1, the ASD and TD groups were balanced based on sex, age, footedness, and socioeconomic status. There was a significant difference in IQ between groups, with TD children having higher scores, although all participants had an IQ in the average range. As expected, children with ASD have higher scores on SRS compared to healthy controls. Considering motor skills, the ASD group had lower scores on MABC2 manual aiming and catching, balance, and total score and lower DCDQ scores with respect to TD.

**Table 1:** Demographic and clinical characteristics of the participants.

	ASD (*n* = 15)	TD (*n* = 16)	*p*-value
Sex	14M/1F	15M/1F	
Footedness	14R/1L	14R/2L	
Age	9.81 ± 1.57	10.01 ± 1.30	0.709
IQ	99.67 ± 23.33	116.06 ± 15.09	**0.026**
PRI	102.47 ± 22.15	113.5 ± 21.02	0.166
SES	65.00 ± 16.15	64.38 ± 16.72	0.917
SRS^∗^	75.27 ± 30.21	26.38 ± 12.38	**0.000**
DCDQ	45.53 ± 12.98	69.69 ± 14.91	**0.000**
MABC 2 – Manual Dexterity	23.33 ± 23.48	35.13 ± 23.65	0.174
MABC 2 – Aiming and Catching	26.37 ± 23.48	57.13 ± 24.82	**0.001**
MABC 2 – Balance	19.20 ± 23.32	49.56 ± 23.11	**0.001**
MABC 2 – Total Score	14.93 ± 13.61	44.94 ± 25.42	**0.000**
ADOS^∗∗^	6.87 ± 1.64	–	
SCQ^∗∗∗^	–	3.13 ± 2.70	

*^∗^raw score; ^∗∗^comparison score; ^∗∗∗^clinical cut off = 15; IQ, Intellectual Quotient; PRI, Perceptual Reasoning Index; SES, Socioeconomic Status; SRS, Social Responsiveness Scale; DCDQ, Developmental Coordination Disorder Questionnaire; MABC 2, Movement Assessment Battery for Children 2; ADOS, Autism Diagnostic Observation Schedule; SCQ, Social Communication Questionnaire. Contrasts in bold are significant at α = 0.05.*

**FIGURE 3:**
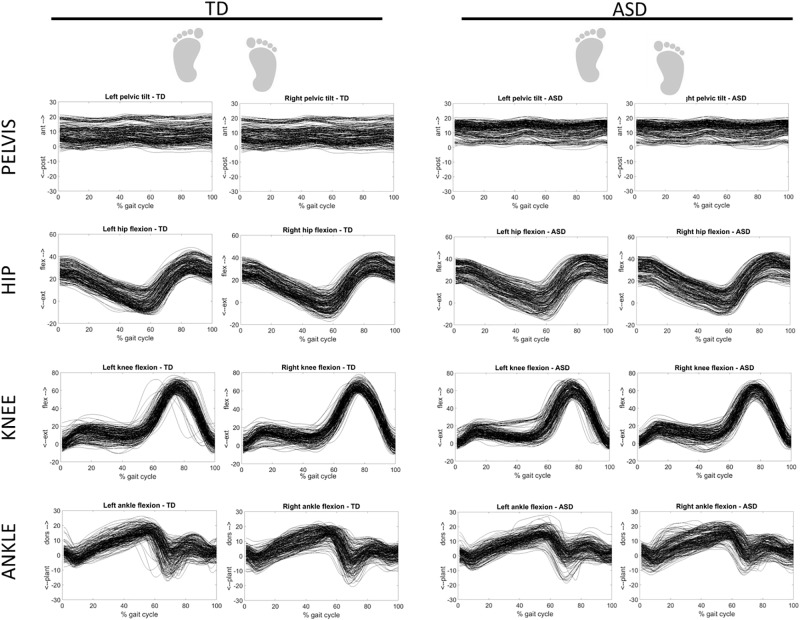
Step-by-step kinematics for the pelvic, hip, knee and ankle joint for TD (left) and ASD (right) subjects. Left and right sides are distinguished. Each waveform represents a normalized (from 0 to 100) step of one subject. All the steps and the subjects are overlapped.

**FIGURE 4:**
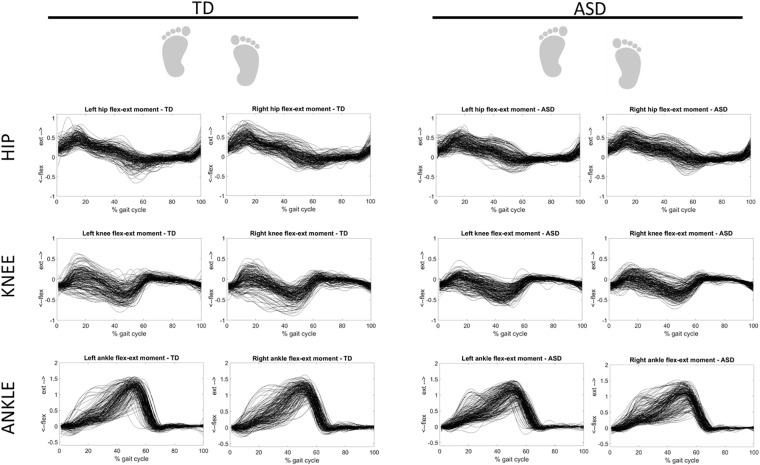
Step-by-step moments for the ankle, knee and hip joint for TD (left) and ASD (right) subjects. Left and right sides are divided. Each waveform represents a normalized (from 0 to 100) step of one subject. All the steps and the subjects are overlapped.

**FIGURE 5:**
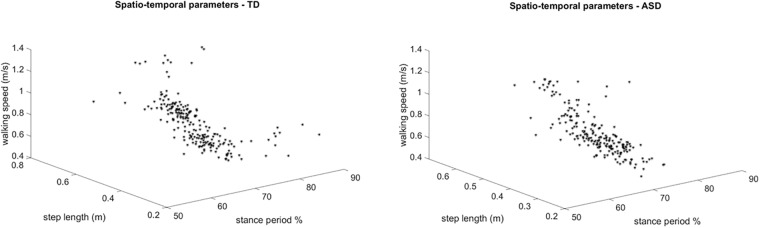
3 dimensional plots of the step-by-step spatiotemporal gait features (step length, stance period and walking speed) for TD (left) and ASD (right) subjects. Each point is referred to a step of one subject.

Figure 3 shows the overlapped and aligned step-by-step kinematics for the pelvic, hip, knee and ankle joint for the TD and ASD group, distinguishing the left from the right side. Figure 4 depicts the lined up step-by-step moments for the hip, knee and ankle joint for the two groups, with the left and the right side divided. Figure 5 shows spatiotemporal parameters (step length, stance period and walking speed) for the two groups. The distribution of data within each subject was normal (Shapiro–Wilk *p*-values > 0.05), thus for every subject a mean value for the left and one for the right side was computed over *N* steps recorded, for each gait parameter.

Statistical tests run to check for differences between the left and right foot detected only one difference at T0 in the ASD group, while no differences were detected in the TD group. Therefore, the mean value between left and right side was computed for each gait feature. Then, normality was verified for all gait features in both groups; thus the ANCOVA test with IQ as covariate was run. The ANCOVA identified significant differences between the ASD group and the TD group at T0. Members of the ASD group showed reduced peak of ankle flexion moment, increased hip flexion at IC, and greater pelvic anteversion (at IC and during the whole gait cycle). Moreover, in the TD group, the step tended to be longer and faster, the knee and hip flexion moments tended to be bigger, and the ROMs of all joints tended to be wider than in the ASD group. Mean gait data of the two groups and the *p*-values of the ANCOVA at T0 are shown in Table 2.

**Table 2:** Gait features of the two groups and differences at T0.

		ASD (*n* = 15)		TD (*n* = 16)		*p*-value of ANCOVA (IQ cov)
		T0	T1	T0	T1
		mean ± *SD*	mean ± *SD*	mean ± *SD*	mean ± *SD*	at T0^&^
**Spatio-temporal**						
**Stance period (% gait cycle)**	**%**	68.19 ± 1.31	68.44 ± 1.11	67.16 ± 2.34	67.77 ± 2.04	0.128
**Step length**	**m**	0.39 ± 0.08	0.39 ± 0.08	0.43 ± 0.07	0.43 ± 0.08	0.371
**Walking speed**	**m/s**	0.79 ± 0.16	0.78 ± 0.16	0.88 ± 0.18	0.88 ± 0.18	0.345
**Kinetics**						
**Peak ankle flexion moment**	**Nm/kg**	1.12 ± 0.22	1.15 ± 0.20	1.27 ± 0.13	1.27 ± 0.18	**0.040**
**Peak knee flexion moment**	**Nm/kg**	0.16 ± 0.07	0.16 ± 0.08	0.22 ± 0.15	0.19 ± 0.14	0.273
**Peak hip flexion moment**	**Nm/kg**	0.57 ± 0.16	0.58 ± 0.19	0.66 ± 0.18	0.71 ± 0.20	0.213
**Kinematics**						
**Ankle flexion initial contact**	**°**	1.67 ± 3.06	1.31 ± 2.76	1.95 ± 2.45	1.47 ± 3.26	0.934
**Ankle flexion ROM^$^ stance**	**°**	19.33 ± 3.22	20.60 ± 3.42	21.23 ± 3.60	23.23 ± 4.80	0.622
**Peak plantarflexion^$$^**	**°**	3.86 ± 3.80	5.73 ± 5.25	6.01 ± 4.07	7.76 ± 5.42	0.302
**Knee flexion initial contact**	**°**	1.66 ± 4.79	0.77 ± 3.76	1.30 ± 4.32	0.97 ± 4.75	0.968
**Knee flexion ROM^$^**	**°**	62.44 ± 4.41	63.58 ± 4.20	64.64 ± 5.08	65.72 ± 5.76	0.453
**Peak knee extension**	**°**	0.71 ± 2.56	1.75 ± 3.36	0.52 ± 4.07	1.07 ± 5.00	0.689
**Hip flexion initial contact**	**°**	30.55 ± 6.07	29.06 ± 6.71	26.76 ± 5.65	25.98 ± 6.47	**0.031**
**Hip flexion ROM^$^**	**°**	34.52 ± 5.56	34.51 ± 4.84	35.50 ± 3.63	36.65 ± 4.12	0.936
**Peak hip extension**	**°**	-1.07 ± 7.11	0.41 ± 6.88	2.26 ± 6.30	3.71 ± 7.01	0.236
**Pelvic tilt initial contact**	**°**	13.40 ± 3.99	12.01 ± 4.43	8.31 ± 5.41	7.15 ± 5.88	**0.007**
**Mean pelvic tilt**	**°**	12.36 ± 4.21	11.03 ± 4.66	8.10 ± 5.39	6.61 ± 5.87	**0.017**

*^$^ROM, range of motion; ^$$^plantarflexion, ankle extension. ^&^p-value of ANCOVA analysis at T0 between ASD and TD. Ranges of motion were all recorded in flexion in the sagittal plane. Contrasts in bold are significant at α = 0.05.*

**FIGURE 6:**
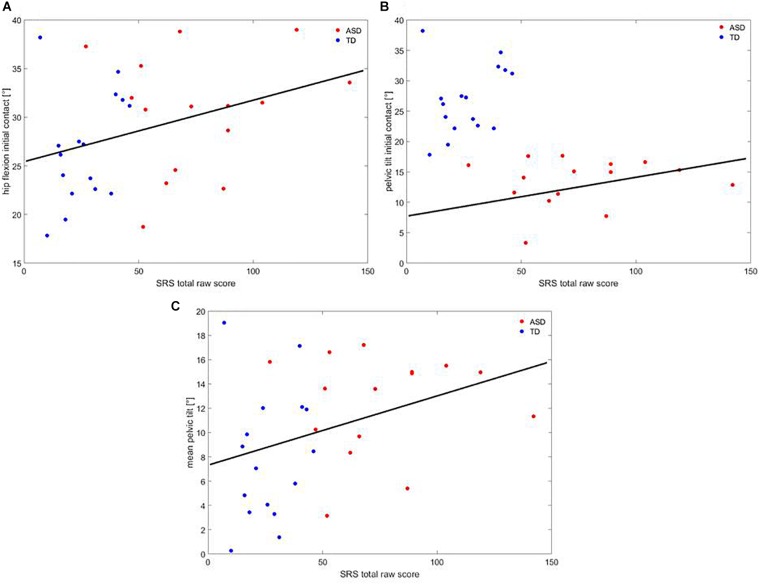
Correlation between **(A)** hip flexion at IC, **(B)** pelvic tilt at IC, **(C)** mean pelvic tilt at IC and SRS. Red dots are children with ASD, blue dots are TD children. The black line represents the regression curve.

A correlation analysis showed that, across participants, SRS correlated with hip flexion at IC (*R* = 0.366; *p* = 0.43), pelvic tilt at IC (*R* = 0.402; *p* = 0.025), and mean pelvic tilt (*R* = 0.359; *p* = 0.047), as shown in Figure 6.

**FIGURE 7:**
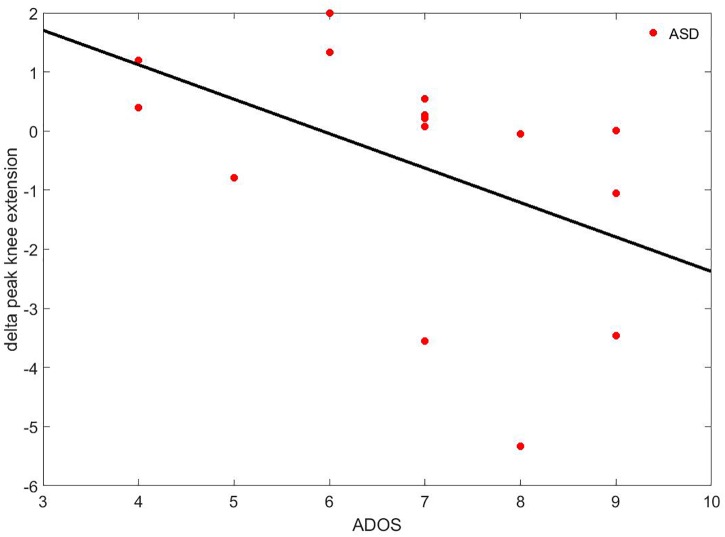
Correlation between the delta peak of knee extension and ADOS. The black line represents the regression curve.

Concerning the differences of the delta parameters (Delta_i-parameter_, see Eq. 1) between the ASD and the TD group, the ANCOVA with IQ as covariate only highlighted one significant difference: the delta peak of knee extension was negative in the ASD group (Δ = −0.55 ± 2.03°) while it was positive in the TD group (Δ = 0.15 ± 0.75°). Moreover, it was correlated to ADOS (rho = −0.637; *p* = 0.011), as shown in Figure 7.

**FIGURE 8:**
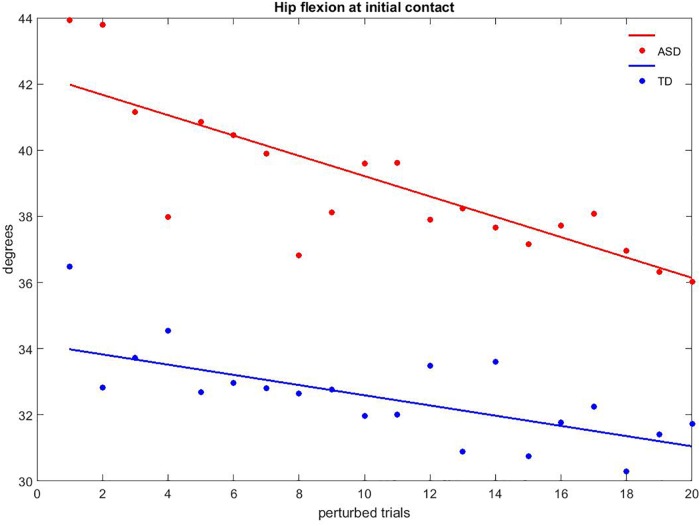
Example of adaptation to discrete perturbation for hip flexion at initial contact. Red dots are children with ASD, and the red line represents the regression curve; blue dots are TD children and the blue line represents the related regression curve.

The fitting of the 20 perturbed trials revealed that more than 60% of the gait variables had a linear trend, with *p*-values lower than 0.05, R2 values between 0.22 and 0.82, and the slope coefficient varying from −0.346 to 0.432. No differences in terms of R2 (ASD: 0.419 ± 0.184, TD: 0.401 ± 0.119; *p* = 0.782) and slope of the curves (ASD: −0.087 ± 0.142, TD: −0.106 ± 0.227; *p* = 0.786) were detected between groups. Figure 8 shows the hip flexion at IC during the perturbed trials as an example of gait adaptation.

Finally, considering the variables that showed a significant fitting in at least one group, it was observed that members of the ASD group had significant changes between T0 and T1 for the walking speed (*p* = 0.017), the peak of ankle flexion moment (*p* = 0.043), the peak of knee extension (*p* = 0.030), and the hip flexion at IC (*p* = 0.011). On the other hand, TD had significant changes between T0 and T1 for the stance period (*p* = 0.021), the peak of hip flexion moment (*p* = 0.016), and the peak of ankle and hip extension (*p* = 0.014 and *p* = 0.036, respectively). In addition, both groups had significant changes for the ROM of ankle flexion (*p* = 0.024 and *p* = 0.007 – ASD and TD), the pelvic tilt at IC (*p* = 0.005 and *p* = 0.001– ASD and TD), and the mean pelvic tilt (*p* = 0.006 and *p* = 0.002 – ASD and TD). Data describing gait features at T1 are reported in Table 2.

## Discussion

The purpose of this study was twofold. The first aim was to evaluate the gait pattern over a treadmill of drug-naïve, school-aged children with ASD compared to a group of peers with typical development using the GRAIL. To date, this is the first study that makes uses this innovative and motivating environment dedicated to gait analysis in a sample of participants with ASD. Second, we intended to investigate, across participants, the motor adaptation of discrete gait perturbations administered with a single, unexpected acceleration of the belt under the dominant leg at toe-off. We hypothesized that children with ASD would present alterations in gait pattern in terms of spatio-temporal, kinetic, and kinematic parameters. Furthermore, we hypothesized that children with ASD would show a reduced or altered adaptation rate.

Regarding the first goal of the study, these findings depicted an altered gait pattern in children with ASD with a range of atypical gait parameters. With respect to kinetic measures, children with ASD showed reduced ground reaction forces at the ankle in accordance with previous studies that used traditional force plates (Calhoun et al., 2011; Pauk et al., 2017). In addition, kinematic data suggested an atypical flexion of the hip, which is in line with Calhoun et al. (2011) and with Pauk et al. (2017), coupled with an anterior displacement of the pelvis at foot IC. Last, children with ASD exhibited a greater pelvic anteroversion throughout the gait cycle, which is in line with the low-functioning subsample of patients investigated by Pauk et al. (2017). As a whole, these data indicated an abnormal gait pattern, where children with ASD tend to augment their own locomotion stability. No between-group differences were found with respect to the spatio-temporal parameters, even though a trend of significance was observed, with patients showing a slightly reduced speed of walking and step length, as well as a tendency to increase the stance phase of the gait cycle. These latter data were in line with previous literature (Nobile et al., 2011). It seemed reasonable that we did not exactly replicate findings from studies on overground gait, since walking on a treadmill can have a trivial but significant effect on several gait parameters, as shown by Van der Krogt et al. (2014). Furthermore, this study found, across groups, a positive relationship between the increase of hip flexion and pelvic anteroversion and lower social abilities, as measured by the SRS.

Regarding the second aim of this study, our results indicated a moderate, significant adaptation to the perturbation in both groups. These findings were also confirmed by the slight modification of unperturbed gait pattern recorded after the discrete gait perturbations. More specifically, TD children presented an augmented knee extension, as if they tended to respond to the perturbation with more flexible joints compared to the slight decrease in members of the ASD group. The decrease of knee extension in children with ASD was significantly related to lower scores on ADOS, suggesting that children who displayed a diminished learning adaptation also presented more severe autistic symptomatology.

Moreover, this work revealed a comparable rate of adaptation between groups. To the best of our knowledge, no studies have yet examined the walking adaptation in children with ASD by means of the type of discrete, single-leg perturbation used in this study. However, despite the similar adaptation rate of the two groups of participants, this study provided intriguing evidence of an atypical pattern of adaptation in children with ASD. Namely, to preserve locomotor stability in response to discrete perturbations, TD children increased the stance period of their gait cycle, whereas children with ASD lowered their walking speed. With respect to forces, TD children increased ground reaction forces at the hip, children with ASD at the ankle. As for adaptation of joints in terms of kinematics, both groups showed modifications at the hip and pelvis. Nonetheless, to cope with the discrete perturbation, TD children augmented the extension of the ankle, whereas children with ASD tended to proximalize the control of inferior limbs, increasing their knee extension. The findings related to gait motor adaptation were in line with the observations deriving from upper limb motor learning and adaptation in people with ASD (Haswell et al., 2009; Izawa et al., 2012; Marko et al., 2015). These studies have consistently reported at least some degree of adaptation to the perturbation in children with ASD, although with slower rates or with different patterns. In addition, the abnormal pattern of motor learning, characterized in children with ASD by an increased sensitivity to proprioceptive error and a decreased sensitivity to visual error, has been directly linked to anomalies in the cerebellum (Marko et al., 2015).

The current study underlined the usefulness of employing standardized measures (MABC2) to identify the presence of motor dysfunction. In line with several other studies (e.g., Grace et al., 2017), children with ASD showed motor impairments at the MABC2, in particular with respect to aiming and catching abilities and balance. On the other hand, this work also demonstrated that standardized measures can be limited when identifying motor impairments significantly related to the clinical symptomatology of the disorders. The innovative multi-sensor platform utilized here for automatic analysis locomotion recognized the data of walking adaptation linked to the severity of autistic core symptoms. If replicated in future studies employing split-belt treadmill systems, this association between objective motor markers and the clinical severity of ASD could inform precision medicine-based approaches to ASD diagnosis and rehabilitation and help researchers move toward a mechanistic description of the disorder.

These results should also be regarded with some limitations in mind. First, this study was limited by its small sample size, although we recruited well-characterized, drug-naïve children with ASD of school age and controls matched by gender and age. A second limitation is that this study was restricted to a group of children with high-functioning ASD, and the findings may not be directly generalizable to children with ASD and intellectual disabilities or to adult patients. Future investigations should also extend this innovative instrumentation of gait analysis to younger populations so that an accurate longitudinal pattern of the locomotor trajectories in ASD can be identified. Although we found that our significant between-groups differences were not dependent on IQ, it could be worthwhile in future studies to replicate the present findings with full IQ-matched TD participants as well. Unfortunately, we did not collect in this study any sensory-based measure, such as Sensory Profile questionnaire, from the participants; therefore, we could not control for a possible effect on the present data of eventual sensory peculiarities in children with ASD. In order to disentangle the possible effect of the visual input on the gait pattern of participants, future extensions of the present work should also directly compare overground gait tested in a conventional gait lab, treadmill walking, and walking on treadmill with an optic flow, in the same cohort of participants. Although we do not consider it appropriate to apply corrections to multiple comparisons because this study was exploratory, these results require replication on a larger scale to verify the generalizability of the novel findings we documented. Last, in the context of precision medicine, new statistical methods for personalized behavioral and kinematic analyses have been recently developed, with particular reference to gait analysis (Torres et al., 2016a,b; Dufek et al., 2017). Along this line, future extension of this study could benefit from this individualized analytical method in order to precisely identify individual differences between children with ASD and controls. In addition, future studies using the GRAIL should eventually consider to compute gait features in frontal and transverse planes based on recent advancements in 3-D motion analysis systems (see, for example, Senanayake and Khoo, 2007; Damit et al., 2017).

## Conclusion

Using the GRAIL, an innovative multi-sensor platform based on immersive VR, we described several peculiarities in the locomotion of children with ASD. In addition, we provided initial, intriguing evidence of an abnormal pattern of adaptation to discrete perturbations in our clinical sample. The reduced mobility of joints was directly related to the gravity of autistic symptomatology. The pattern of these findings could be compatible with a possible involvement of the cerebellum. Future research based on both neuroimaging and the detailed kinematic characterization of gait could help better understand the neural correlations of the motor system in people who have ASD. Furthermore, the possibility to systematically manipulate the visual and proprioceptive feedback ensured by GRAIL will allow researchers to test whether, such as for upper limb motor learning, children with ASD show a greater learning from proprioceptive errors rather than visual errors, with an opposite pattern compared to typical motor learning. Finally, the VR environment offered a highly stimulating and rewarding setting for children with ASD. This innovative aspect represents a fascinating perspective not only in terms of quantitative motor evaluation but in terms of developing educational programs for the rehabilitation of children with ASD beyond the motor domain.

## Author Contributions

EB, CC, SBC, and ACr conceived, designed, and drafted this work. EB and ACe conceived and designed the gait paradigm. EB, CC, SBC, and ACe performed the clinical and experimental evaluation of all participants. All authors critically revised, and approved the final version of and agreed to be accountable for this work.

## Conflict of Interest Statement

The authors declare that the research was conducted in the absence of any commercial or financial relationships that could be construed as a potential conflict of interest.
